# Anti-Fibrotic Effect of Oleamide Identified from the *Moringa oleifera* Lam. Leaves via Inhibition of TGF-β1-Induced SMAD2/3 Signaling Pathway

**DOI:** 10.3390/ijms26073388

**Published:** 2025-04-04

**Authors:** Chavisa Khongpiroon, Watunyoo Buakaew, Paul J. Brindley, Saranyapin Potikanond, Krai Daowtak, Yordhathai Thongsri, Pachuen Potup, Kanchana Usuwanthim

**Affiliations:** 1 Cellular and Molecular Immunology Research Unit, Faculty of Allied Health Sciences, Naresuan University, Phitsanulok 65000, Thailandkraid@nu.ac.th (K.D.); yordhathait@nu.ac.th (Y.T.); pachuenp@nu.ac.th (P.P.); 2 Department of Microbiology, Faculty of Medicine, Srinakharinwirot University, Bangkok 10110, Thailand; watunyoo@g.swu.ac.th; 3 Department of Microbiology, Immunology and Tropical Medicine, School of Medicine & Health Sciences, George Washington University, Washington, DC 20037, USA; pbrindley@gwu.edu; 4 Department of Pharmacology, Faculty of Medicine, Chiang Mai University, Chiang Mai 50200, Thailand; saranyapin.p@cmu.ac.th

**Keywords:** *Moringa oleifera* Lam., oleamide, liver fibrosis, TGF-β/SMAD2/3 pathway, anti-fibrotic effect

## Abstract

*Moringa oleifera* (MO) is a prominent plant in traditional medicine, widely recognized for its phytochemicals with anti-inflammatory properties. Liver fibrosis characterized by chronic inflammation and excessive extracellular matrix deposition may benefit from the therapeutic properties of MO. This report focuses on the potential of oleamide (OLA), a bioactive compound identified from MO, in mitigating liver fibrosis. The anti-fibrotic effects of OLA were evaluated by assessing the production of pro-inflammatory cytokines, gelatinase activity and the expression of genes and proteins associated with the TGF-β/SMAD2/3 pathway. The LX-2 human hepatic stellate cell line, in conjunction with TGF-β1, was employed to model fibrotic conditions. OLA treatment significantly reduced the production of pro-fibrotic effectors in the activated LX-2 cells. Molecular docking analysis demonstrated a high binding affinity of OLA to key proteins in the TGF-β/SMAD2/3 pathway, while qRT-PCR and Western blotting revealed that OLA suppressed the expression of COL1A1, COL4A1, SMAD2, SMAD3, SMAD4, MMP2, MMP9, ACTA2 and TIMP1. These findings indicate that OLA effectively attenuates the pro-inflammatory responses induced by TGF-β1 and inhibits the activation of LX-2 cells. Collectively, OLA holds significant potential as a therapeutic agent for the prevention and treatment of liver fibrosis via the modulation of the TGF-β/SMAD2/3 signaling pathway.

## 1. Introduction

The liver is a vital organ located in the upper right abdomen. The liver performs important functions in the body, including metabolism, immunity, digestion, detoxification and vitamin storage [1]. Liver diseases can be classified into different stages. The initial phase of liver disease is hepatitis, characterized by the inflammation of the liver. This condition is primarily attributed to viral infections, such as hepatitis B and C, or chronic alcohol consumption, both of which induce hepatic damage through immune system activation and oxidative stress mechanisms [2]. Chronic liver inflammation may lead to the accumulation of lipid deposits within hepatocytes, resulting in a pathological condition termed liver steatosis or fatty liver disease. This condition emerges when the homeostasis of lipid acquisition and removal within hepatocytes is disrupted, often due to metabolic dysfunction or exposure to toxic agents [3]. If left untreated, liver steatosis may progress to fibrosis, a pathological state characterized by the excessive deposition of extracellular matrix components, particularly collagen.

Initially an adaptive wound-healing response to chronic liver injury, fibrosis turns pathogenic when the normal tissue architecture is not restored by repairing mechanisms [4]. Advanced fibrosis can further progress to cirrhosis, a severe condition defined by extensive hepatic scarring and significant disruption of liver tissue architecture. Cirrhosis results in profound impairment of liver function and predisposes individuals to life-threatening complications, including portal hypertension and liver failure [5]. Cirrhosis represents the most significant risk factor for the development of hepatocellular carcinoma (HCC), the predominant form of primary liver malignancy. Persistent inflammation in cirrhotic tissue facilitates genetic mutations and DNA damage during hepatocyte regeneration, thereby increasing the likelihood of neoplastic transformation [6]. The fibrotic and cirrhotic microenvironments are characterized by pro-tumorigenic conditions, including hypoxia, oxidative stress and the activation of key signaling pathways such as transforming growth factor-beta (TGF-β). These factors promote cellular proliferation, survival and migration while simultaneously impairing immune surveillance [5]. Liver inflammation can indeed be treated and mitigated [7]. However, liver cirrhosis, characterized by extensive hepatic tissue damage, may not be reversible to its normal physiological state [8]. Therefore, it is important to treat liver inflammation before it progresses to the stage of liver cirrhosis [9]. Liver fibrosis is a response to chronic liver injury that involves the accumulation of extracellular matrix (ECM) components, such as type I collagen, ACTA2 (Actin Alpha 2, Smooth Muscle), fibronectin and hyaluronic acid [10]. This accumulation occurs due to an imbalance in the synthesis and degradation of ECM, which disrupts normal liver function [11]. TGF-β is a key signaling molecule that plays a critical role in liver fibrosis by promoting fibrogenesis. It stimulates the activation and proliferation of hepatic stellate cells (HSCs), which are normally quiescent cells in the liver. Upon activation, HSCs transdifferentiate into myofibroblast-like cells and secrete excessive extracellular matrix (ECM) components, contributing to the accumulation of scar tissue. This excessive ECM deposition is a hallmark of liver fibrosis. Furthermore, TGF-β inhibits ECM degradation, thereby enhancing fibrosis progression. In addition to its effects on HSCs, TGF-β is involved in the modulation of immune cells, driving inflammatory responses that further exacerbate liver injury and fibrosis. These immune cells, including macrophages and lymphocytes, are recruited to the liver, where they release pro-inflammatory cytokines that promote HSC activation and worsen fibrotic processes. Given these roles, targeting the activation of HSCs and inhibiting the TGF-β/mothers against the decapentaplegic homolog 2 and 3 (SMAD 2/3) signaling pathway are promising strategies for treating liver fibrosis. By blocking TGF-β signaling, it is possible to reduce HSC activation, limit ECM accumulation and attenuate the inflammatory environment, ultimately slowing or preventing the progression of liver fibrosis. This approach is being actively explored as a therapeutic avenue to address liver diseases associated with excessive fibrosis, including cirrhosis and non-alcoholic steatohepatitis (NASH) [12,13,14,15,16]. Some researchers are investigating the use of natural extracts to target these pathways as potential treatments for liver fibrosis [17,18,19].

*Moringa oleifera* Lam. (MO), commonly known as the drumstick tree or miracle tree, is a fast-growing, drought-resistant plant native to northern India and widely cultivated in tropical and subtropical regions [20]. Renowned for its therapeutic properties, it is rich in essential nutrients, including vitamins A, B and C; minerals; and proteins, and contains bioactive compounds such as flavonoids, isothiocyanates and phenolic acids [21]. These contribute to its medicinal applications, which include anti-microbial, anti-inflammatory and antioxidant effects that mitigate oxidative stress- and inflammation-related disorders [22,23]. Wisitpongpun et al. (2020) established oleamide (OLA) as one of several bioactive compounds from MO leaves. This study revealed that OLA exhibited anticancer activity by causing cell cycle arrest and initiating apoptosis [24]. OLA, an organic compound derived from oleic acid, occurs naturally. Research has explored its multifaceted properties for health benefits, including its impact on sleep and cognitive activities [24,25], memory [25] and anti-inflammatory properties [26,27]. Additionally, OLA has been investigated for its potential to prevent and treat diseases characterized by inflammatory components [25,28]. However, research is limited on the potential anti-liver fibrosis effects of OLA, a specific compound in MO. The present study investigated the effectiveness of OLA identified from MO for the treatment of hepatic fibrosis. TGF-β1-activated human hepatic stellate cells exposed to OLA were studied in a model for liver fibrosis. OLA markedly inhibited liver fibrotic markers, such as pro-inflammatory cytokines, ACTA2, TIMP1, COL1A1, COL4A1, SMAD2, SMAD3, SMAD4, MMP2 and MMP9. Based on the TGF-β/SMAD2/3 signaling pathway, the results showed that OLA has potential as a therapeutic drug for liver fibrosis.

## 2. Results

### 2.1. OLA Suppresses Activation of Hepatic Stellate Cells (HSCs) via TGF-β1

**Figure 1:**
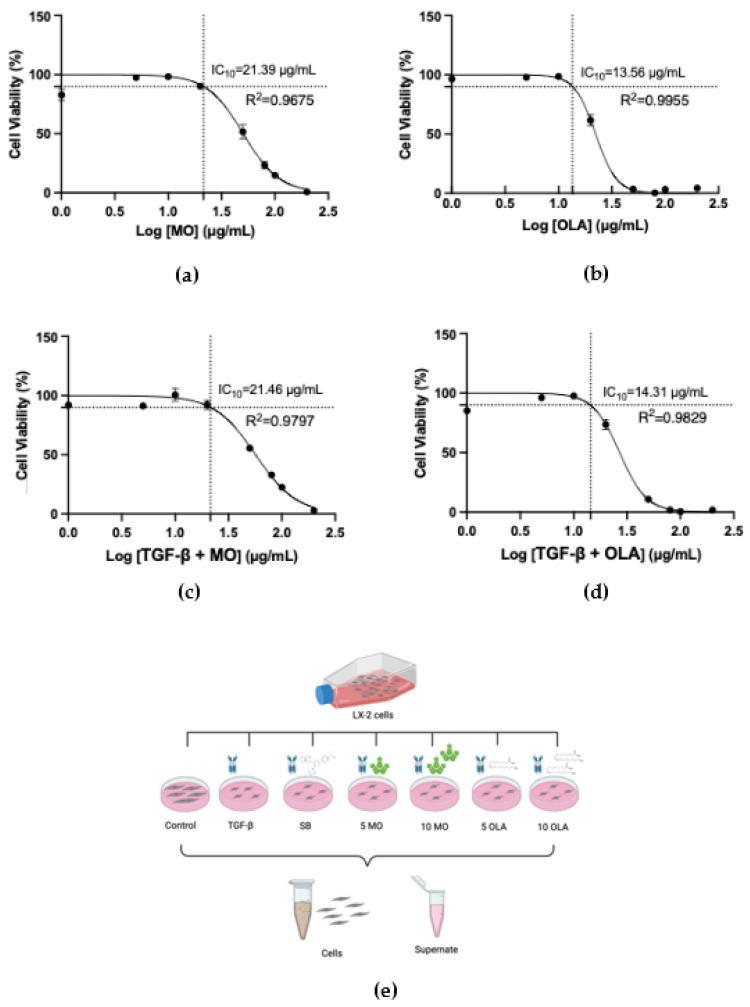
The cytotoxicity of MO and OLA in the concentration range of 0–200 μg/mL, as assessed by the resazurin reduction assay. (**a**) MO crude extract on normal LX-2 cells; (**b**) OLA on normal LX-2 cells; (**c**) MO crude extract on LX-2 cells induced by TGF-β; (**d**) OLA on LX-2 cells induced by TGF-β1; (**e**) a schematic diagram of the experimental design. Control: untreated; TGF-β: transforming growth factor-beta 10 ng/mL; SB:SB431542 (4-(4-(benzo[d][1,3]dioxol-5-yl)-5-(pyridin-2-yl)-1H-imidazol-2-yl)benzamide)10 mM; 5 MO: *Moringa oleifera* leaf extract 5 μg/mL; 10 MO: MO 10 μg/mL; 5 OLA: oleamide 5 μg/mL; 10 OLA: oleamide 10 μg/mL.

Crude extract from MO was prepared using a solvent mixture of Tween20 and dimethyl sulfoxide (DMSO), and OLA was dissolved in this solvent. HSC activation is a critical process during hepatic fibrosis. This study used the human LX-2 cell line, which is characterized as an HSC cell line. Firstly, the cytotoxicity of MO crude extract and OLA in the concentration range of 0–200 μg/mL on untreated cells and LX-2 cells induced by TGF-β1 was determined by the resazurin reduction assay. After 48 h incubation, concentrations ≤10 μg/mL of MO crude extract and OLA were found not to be toxic to >90% (IC_10_) of the cells (Figure 1a–d). We selected two concentrations of MO crude extract and OLA (5, 10 μg/mL) for further investigation. Figure 1e presents a schematic diagram of the experimental design.

**Figure 2:**
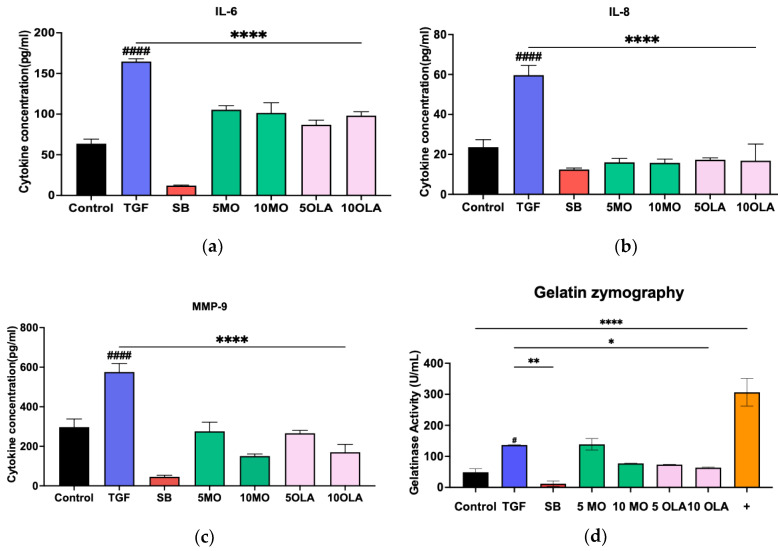
The cytokine production of (**a**) IL-6, (**b**) IL-8 and (**c**) MMP-9 and (**d**) gelatin zymography. Control: untreated; TGF-β: transforming growth factor-beta 10 ng/mL; SB:SB431542 10 mM, MO: *Moringa oleifera* Lam. leaves extract; 5 MO: 5 μg/mL of MO; 10 MO: 10 μg/mL of MO; OLA: oleamide; 5 OLA: OLA 5 μg/mL; 10 OLA: OLA 10 μg/mL; +: positive control. Data are represented as mean ± SD. ^####^ *p* < 0.0001 compared to control, * *p* = 0.0306, ** *p* = 0.0032 and **** *p* ≤ 0.0001 compared to the TGF-β1-treated group; ns: not significant.

The initial investigation explored whether MO crude extract and OLA ameliorate hepatocyte injury and liver fibrosis on LX-2 cells by the analysis of cytokine and ECM production (IL-6, IL-8 and MMP-9). LX-2 cells induced by TGF-β secreted IL-6, IL-8 and MMP-9 at higher levels than LX-2 cells without TGF-β stimulation. In contrast, when treated with SB431542 (a specific small-molecule inhibitor that targets the TGF-β), crude MO and OLA induced less secretion of IL-6, IL-8 and MMP-9 than without SB431542 treatment (Figure 2a–c). Moreover, we determined the metalloproteinase activity by the gelatin degradation assay. LX-2 cells induced by TGF-β show significantly increased gelatinase activity compared with LX-2 cells without TGF-β stimulation. In contrast, when treated with crude MO and OLA, LX-2 cells show less gelatinase activity than non-treated cells (Figure 2d).

### 2.2. OLA Suppresses Fibrotic Markers and Inhibits HSCs Activation by Blocking the SMAD Signaling Pathway at the Gene Expression Level

**Figure 3:**
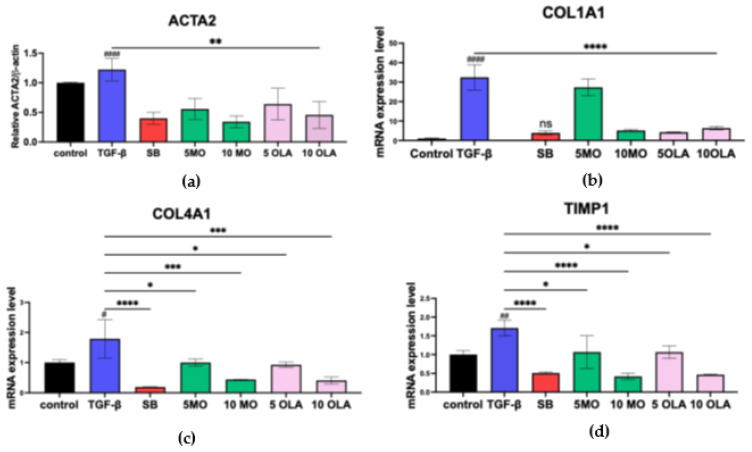

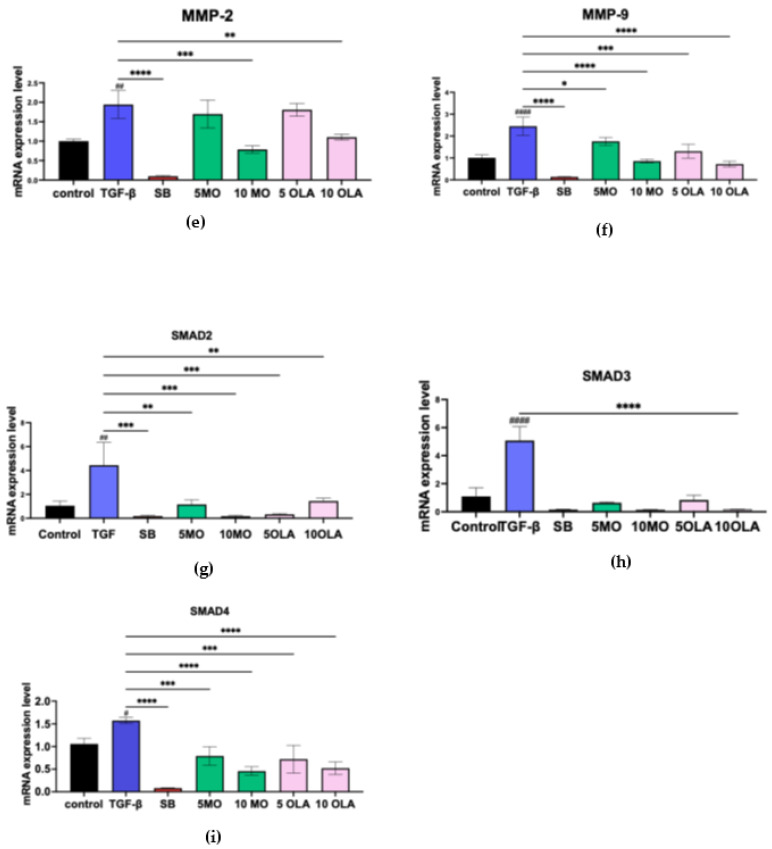
Effects of OLA identified from the MO leaves on mRNA expression. The expressions of (**a**) *ACTA2*, (**b**) *COL1A1,* (**c**) *COL4A1,* (**d**) *TIMP1,* (**e**) *MMP2,* (**f**) *MMP9,* (**g**) *SMAD2,* (**h**) *SMAD3* and (**i**) *SMAD4* were assessed using real-time qRT-PCR. *GAPDH* served as the internal control. Control: untreated; TGF-β: transforming growth factor-beta 10 ng/mL; SB:SB431542 10 mM, 5 MO: *Moringa oleifera* leaf extract 5 μg/mL; 10 MO: MO 10 μg/mL; 5 OLA: oleamide 5 μg/mL; 10 OLA: OLA 10 μg/mL. Data are represented as mean ± SD. # *p* < 0.05, ## *p* < 0.01, #### *p* < 0.0001 compared to control, * *p* = 0.0240, ** *p* = 0.0034, *** *p* = 0.0005 and **** *p* ≤ 0.0001 compared to the TGF-β1-treated group; ns: not significant.

Based on the observation that OLA suppresses HSCs activation via TGF-β1, we hypothesized that the extract also suppresses informative fibrotic markers at the gene expression level. To test this hypothesis, the mRNA expression levels of ACTA2, COL1A, COL4A1, TIMP-1, MMP-2 and MMP-9 were determined by real-time qRT-PCR using GAPDH as an internal control. After incubation for 48 h with 10 ng/mL TGF-β1, the expression of all selected genes had markedly increased, as seen in Figure 3a–f, compared to untreated cells. Notably, the extract at 10 μg/mL could suppress the expression of all selected genes. These results suggest that OLA initially suppresses fibrotic markers at the transcriptional level. Therefore, we evaluated whether OLA exerts its anti-fibrotic ability through the suppression of SMAD2/3 induced by TGF-β1. Cells treated with TGF-β1 showed increases in SMAD2, SMAD3 and SMAD4 mRNA expression compared to untreated cells, as seen in Figure 3g–i.

### 2.3. OLA Suppresses Fibrotic Markers via the SMAD2/3 Signaling Pathway at Translational Levels

**Figure 4:**
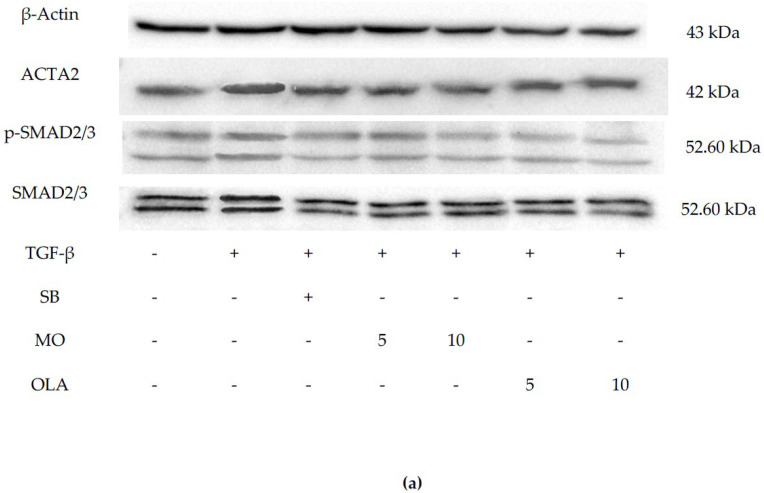

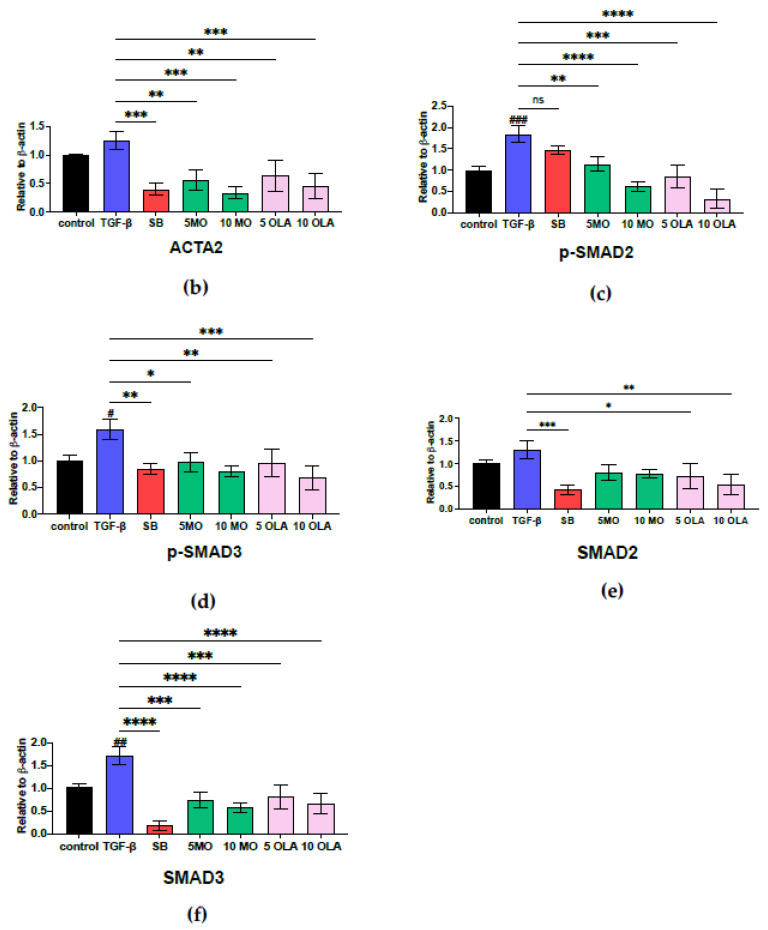
Effects of MO and OLA on ACTA2, p-SMAD2/3 and SMAD2/3 protein production. The quantitative protein production of ACTA2, p-SMAD2/3 and SMAD2/3 was measured by Western blotting (**a**). The bar graphs represent the relative expression of these proteins after normalization to β-actin (**b**–**f**). Control: untreated; TGF-β: transforming growth factor-beta 10 ng/mL; SB:SB431542 10 mM, 5 MO: Moringa oleifera leaf extract 5 μg/mL; 10 MO: MO 10 μg/mL; 5 OLA: oleamide 5 μg/mL; 10 OLA: OLA 10 μg/mL. Data are represented as mean ± SD. # *p* < 0.0001 ## *p* < 0.01, ### *p* < 0.001 compared to control, * *p* ≤ 0.1, ** *p* ≤ 0.01, *** *p* ≤ 0.001 and **** *p* ≤ 0.0001 compared to the control * *p* < 0.0001 indicates statistical significance compared to the TGF-β1-treated group; ns: not significant.

Following the observed reduction in the expression of key fibrosis-related and SMAD signaling genes ACTA2, COL1A1, COL4A1, TIMP-1, MMP-2, MMP-9, SMAD2, SMAD3 and SMAD4 after OLA treatment, we confirmed these findings by analyzing protein levels using immunoblot analysis. In untreated cells, the protein levels of ACTA2, p-SMAD2/3 and SMAD2/3 were low. However, TGF-β1 treatment markedly induced the production of these proteins, as shown in Figure 4a. Consistent with expectations, OLA significantly suppressed TGF-β1-induced production of ACTA2, p-SMAD2/3 and SMAD2/3 in a concentration-dependent manner, as demonstrated in Figure 4b–f.

### 2.4. In Silico Molecular Docking Analysis

**Figure 5:**
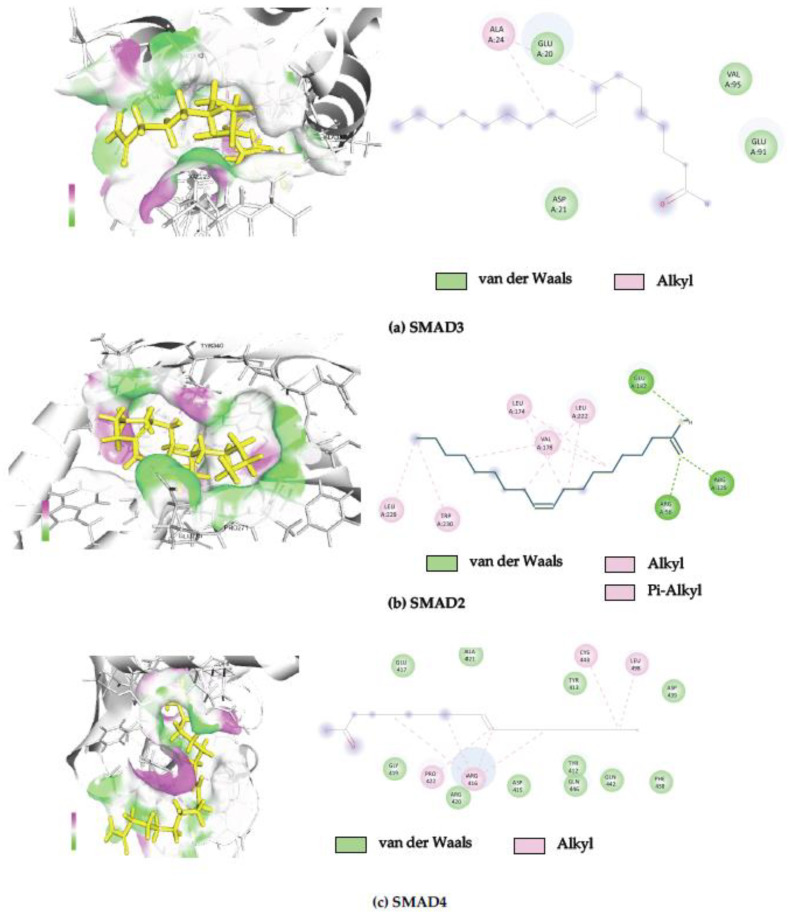

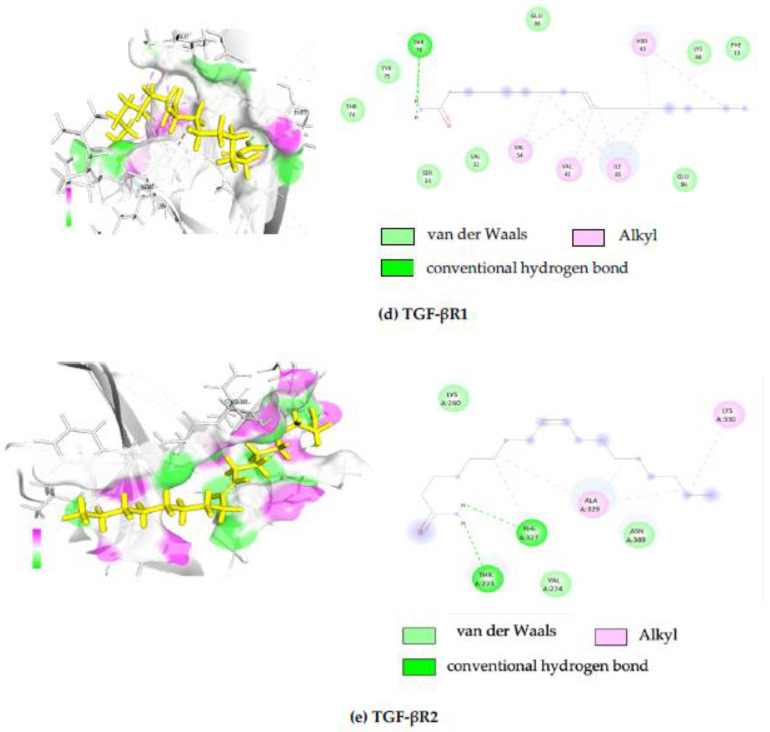
Three-dimensional and two-dimensional images showing the binding of OLA to protein in the SMAD signaling pathway. (**a**) SMAD3, (**b**) SMAD2, (**c**) SMAD4, (**d**) TGF-βR1 and (**e**) TGF-βR2.

Binding interactions between OLA and potential protein targets were examined using the cavity detection-guided blind docking capabilities of the SWISS-dock web-based platform. Findings including Autodock Vina binding scores and the amino acids involved in the interactions are detailed in Table 1. A visualization of the protein–ligand interactions in 2D and 3D formats is shown in Figure 5. Of particular interest, OLA displayed favorable binding scores (−6 to −8 Kcal/mol) with target proteins known to participate in the SMAD signaling pathway.

There are many proteins that bind in the SMAD signaling pathway, and molecular docking provides insights into the specific interactions between OLA and proteins in the SMAD2/3 pathway. This understanding can facilitate the unraveling of the molecular mechanisms underlying cellular signaling, as seen in Table 1. The structural information (2D and 3D) of OLA protein docking combination can be seen in Figure 5a–e.

## 3. Discussion

Liver fibrosis, considered a precursor to irreversible cirrhosis, results from persistent liver injury and predisposes individuals to severe complications such as portal hypertension, hepatic encephalopathy and hepatocellular carcinoma. Current therapeutic approaches primarily focus on managing these underlying causes and implementing lifestyle modifications, as there is no FDA-approved medication specifically designed to treat liver fibrosis. However, recent advancements in research offer promising prospects. In March 2024, the FDA approved resmetirom (Rezdiffra) [29] for treating adults with noncirrhotic non-alcoholic steatohepatitis (NASH) with moderate-to-advanced fibrosis, marking a significant step in targeted therapy. Additionally, Akero therapeutics reported that efruxifermin (EFX), an FGF21-targeting drug, demonstrated a 39% reversal of cirrhosis in a Phase 3 study compared to only 15% in the placebo group [30], further emphasizing the potential of novel therapeutic interventions. Other drugs, such as cenicriviroc [31], have also shown promise in clinical trials, highlighting the ongoing exploration of anti-fibrotic agents.

At the molecular level, hepatic stellate cell (HSC) activation plays a pivotal role in the pathogenesis of liver fibrosis, with transforming growth factor beta-1 (TGF-β1) serving as a key activator through both the canonical (SMAD-dependent) and non-canonical (non-SMAD) pathways. The TGF-β1/SMAD signaling cascade, a well-documented driver of HSC activation, is initiated when TGF-β1 binds to its specific receptor, TGF-β type II receptor (TβRII), leading to the recruitment and phosphorylation of TGF-β type I receptor (TβRI). This cascade results in the phosphorylation of Smad2 and Smad3, which then form a complex with Smad4 and translocate to the nucleus, where they regulate the transcription of fibrosis-related genes. Several key genes, including *ACTA2, COL1A1, COL4A1, TIMP1, MMP2* and *MMP9*, are involved in extracellular matrix remodeling and are influenced by TGF-β signaling.

The present study demonstrated that activation of the TGF-β/SMAD signaling pathway in HSCs leads to the upregulation of these fibrosis-associated genes, along with increased secretion of inflammatory markers such as IL-6 and IL-8, as well as matrix metalloproteinases. However, treatment with *Moringa oleifera* Lam. (MO) leaf extract and oleamide (OLA) showed the suppression of fibrosis-related markers at both the mRNA and protein levels. This suggests that MO and OLA interferes with HSC activation by disrupting the TGF-β/SMAD signaling cascade, particularly by downregulating Smad2 and Smad3 production. The findings agree with those of previous studies that investigated how natural substances might be used to target these molecular pathways and prevent liver fibrosis, including *Salacia chinensis* L. Stem extract [32], 1-Phenyl-2-Pentanol identified from MO [33] and β-Citronellol identified from *Citrus histrix* DC [34]. Additionally, hepatic fibrosis was decreased by anti-fibrosis molecules such as fluorofenidone, which blocked TGFβ1-1/SMAD and MAPK signaling [35]. Praziquantel dramatically raised the expression of Smad7 in the HSCs of mice with liver fibrosis while decreasing the synthesis of collagen [36]. Furthermore, the key fibrotic markers α-SMA and COL1A1 were significantly decreased by glyceraldehyde (GA), although GA substantially increased the formation of reactive oxygen species (ROS) and markedly raised ERK and JNK phosphorylation [37]. Through the activation of HSCs, ROS and oxidative stress have played a crucial role in the start of fibrogenesis. Since antioxidants can decrease the formation of ROS, they are becoming more popular as potential anti-fibrotic therapy. Thus, a number of antioxidants, such as S-adenosyl-L-methionine (SAMe), silymarin, phosphatidylcholine, resveratrol, quercetin, N-acetylcysteine (NAC), s-allylcysteine (SAC), oroxylin A, methyl ferulic acid (MFA) and vitamin E, are under investigation in clinical trials with encouraging outcomes. Quercetin, daidzein, resveratrol, cyperus, curcumin, thymol, apigenin, rice bran oil, red yeast rice golden berry and NAC can effectively protect against liver damage and inhibit stellate cell activation in both animal models and cell cultures [38]. Since MO and OLA have antioxidant properties, they likewise may be valuable for the treatment of liver fibrosis.

Furthermore, molecular docking has provided insights into potential interactions between OLA and key signaling proteins, including TGF-βR1, TGF-βR2, Smad2, Smad3 and Smad4. Comparative binding energy analysis using the SwissDock platform within the Chimera software 1.19 framework suggests that OLA binds to these proteins at sites overlapping with known ligands, potentially inhibiting their activity. Predicted binding sites were further analyzed using BIOVIA Discovery Studio Visualizer software, version 21.1.0.20298 reinforcing the hypothesis that OLA disrupts SMAD signaling by interacting with Smad2, Smad3 and Smad4. These in silico findings were corroborated by Western blot analysis, which confirmed that OLA effectively attenuates the transcriptional expression of fibrosis-associated proteins, thereby interfering with the canonical TGF-β1-driven signaling cascade. This positions OLA as a promising candidate for further investigation into the development of novel anti-fibrotic agents. However, in vivo and clinical studies remain essential to validate these findings and fully elucidate the pharmacological effects and mechanisms of action of OLA in the context of liver fibrosis. While liver fibrosis poses significant health risks due to its asymptomatic progression and severe complications, advancements in targeted therapies, along with a deeper understanding of molecular mechanisms, offer hope for more effective treatments.

## 4. Materials and Methods

### 4.1. Oleamide Identified from the Moringa oleifera Lam. Leaves

Using the LC-ESI-QTOF-MS/MS chromatogram, the bioactive components in the crude ethyl acetate of *Moringa oleifera* Lam. (MO) leaves were identified, and oleamide (OLA) or Cis-9,10-octadecenamide was discovered (Figure S1). Our earlier study [24] detailed the process for MO extraction and bioactive component identification (Supplementary Materials). The ethyl acetate crude extract of MO leaves was utilized to evaluate anti-liver fibrosis effects in this study. For comparison, OLA (Sigma Aldrich, St. Louis, MO, USA) was purchased and included in the experiments. Both the crude extract and oleamide were dissolved in a solvent mixture of DMSO and Tween 80 (1:1) to enhance solubility in water. In the cell culture experiments, the final concentration of the dissolving solvent residue was kept below 0.5% at the highest tested concentrations of both compounds.

### 4.2. Cell Culture

The human HSC cell line, LX-2 cells, was cultured in Dulbecco’s modified Eagle’s medium (DMEM; Gibco, Thermo Fisher Scientific, Waltham, MA, USA), which was supplemented with 2% fetal bovine serum (Gibco, Carlsbad, CA, USA), 1% penicillin/streptomycin (Gibco–Thermo Fisher Scientific, MA, USA) and 10 millimolar HEPES. The cells were maintained in a humidified incubator set to 37 °C with 5% CO₂. This cell line was graciously provided by Assoc. Prof. Saranyapin Potikanond, M.D., Ph.D., from the Department of Pharmacology, Chiang Mai University, Thailand.

In each experiment, fibrosis was induced in the cells using TGF-β (Abcam AB50036, Cambridge, UK) at a concentration of 10 ng/mL. The cells were then treated with SB431542 (4-(4-(benzo[d][1,3]dioxol-5-yl)-5-(pyridin-2-yl)-1H-imidazol-2-yl)benzamide) (Sigma Aldrich, St. Louis, MO, USA) as a positive control. Additionally, OLA was used for treatment at concentrations of 5 µg/mL and 10 µg/mL.

### 4.3. Cell Viability Assay

To establish the optimal treatment concentrations of crude MO and OLA on LX-2 cells, a resazurin reduction cytotoxicity assay was conducted [33]. LX-2 cells were seeded (2 × 10^4^ cells/well) in a 96-well plate and treated with varying concentrations of MO and OLA. Following 24 h of incubation at 37 °C, resazurin (20 µg/mL, Millipore Sigma, Burlington, MA, USA) was added, and fluorescence was measured after 4 h (excitation/emission: 560/590 nm) using an EnSpire^®^ Multimode microplate reader (PerkinElmer, Waltham, MA, USA). GraphPad Prism version 8 (GraphPad Software, La Jolla, CA, USA) was used to determine inhibitory concentrations (ICs). The IC_10_ value was selected as the maximum safe concentration for further experimental use.

### 4.4. Enzyme-Linked Immunosorbent Assay (ELISA)

LX-2 cells were cultured at a density of 1 × 10⁵ cells/mL. They were then induced with TGF-β and treated with OLA for 24 h. Thereafter, the supernatant was collected, and the levels of IL-6, IL-8 and MMP-9 were measured using the human IL-6 ELISA pair set kit (cat. SEKB10395), human IL-8 ELISA pair set kit (cat. KA00006) and human MMP-9 ELISA pair set kit (cat. SEKA10327) (Sino Biological Inc., Chesterbrook, PA, USA) according to the manufacturer’s instructions. The absorbance signals were detected using a Varioskan LUX Multimode Microplate Reader (Thermo Fisher Scientific, Waltham, MA, USA).

### 4.5. Gelatin Degradation Assay

The gelatin degradation assay is an alternative to traditional zymography for detecting gelatinase activity. It employs a highly quenched gelatin substrate that, when cleaved by gelatinase, releases a fluorophore detectable with a microplate reader. This method is straightforward, substrate-specific and adaptable for high-throughput screening. However, it does not differentiate between specific matrix metalloproteinases (MMPs), such as MMP-2 and MMP-9, unless these enzymes are pre-isolated. Each unit (1 U) of gelatinase is defined as the amount required to release 1 pmol of fluorescein per minute under assay conditions. The kit was purchased from Abcam AB234057 (Cambridge, UK) and used according to the manufacturer’s protocol.

### 4.6. Real-Time Quantitative Reverse Transcription (qRT-PCR)

The medium was removed, and TRIzolTM reagent (300 μL/well) was added, followed by an incubation period of 15 min. Chloroform (50 μL/well) was then added, and the mixture was vortexed twice before centrifugation at 12,000 RPM for 15 min. The supernatant was transferred to a new tube, and isopropanol (200 μL) was added. After mixing, the solution was centrifuged at 12,000 RPM for 10 min. The supernatant was discarded, and 75% ethanol (200 μL) was added to the pellet, followed by centrifugation at 12,000 RPM for 10 min. The supernatant was discarded, and the pellet was air-dried and stored at −20 °C.

For the synthesis of cDNA, 40 μL of RNase-free water was added to each RNA microtube, followed by two rounds of vortexing and centrifugation. Next, 4 μL of the Tetro cDNA Synthesis Kit (Meridian Life Sciences, Cincinnati, OH, USA) reagent was added to a new PCR reaction tube, and 6 μL of RNA was added. After vortexing and centrifugation, the cDNA synthesis reaction was performed. In real-time qRT-PCR, for the measurement of pro-inflammatory cytokines, the reagents were prepared, including primers for *ACTA2, COL1A1, COL4A1, TIMP1, MMP2, MMP9, SMAD2, SMAD3* and *SMAD4*. cDNA from the experiments (3 μL) and SensiFASTTM SYBR^®^ No-ROX Kit reagent (5 μL) were added to a PCR tube, vortexed and centrifuged for 5 s. The reaction was carried out on a CFX96 Touch real-time polymerase chain reaction detection system (Bio-Rad Laboratories, Inc., Hercules, CA, USA). Thermal cycling included of 1 min of polymerase activation at 95 °C, followed by 45 cycles of denaturation at 95 °C for 15 sec, followed by 1 min of annealing and extension at 60 °C. The housekeeping gene was human actin beta (*ACTB*). The data were analyzed using the 2^−ΔΔCT^ approach to normalize gene expression. Primer sequences are shown in Table S1.

### 4.7. Molecular Docking

A molecular docking experiment was conducted to evaluate the interactions between OLA and selected proteins involved in hepatic stellate cell (HSC) activation, a critical process in liver fibrosis. Three-dimensional protein structures associated with HSC activation in hepatic fibrosis were retrieved from the RCSB Protein Data Bank (RCSB PDB) [39,40].

The crystal structures of candidate proteins in the TGF-β/SMAD signaling pathway were retrieved from public databases, including transforming growth factor-beta (TGF-β) receptor type 1 (PDB ID: 5E8X), transforming growth factor-beta (TGF-β) receptor type 2 (PDB ID: 5E8V), mothers against decapentaplegic homolog 2 (SMAD2) (PDB ID: 6YIA), mothers against decapentaplegic homolog 3 (SMAD3) (PDB ID: 6YIB) and mothers against decapentaplegic homolog 4 (SMAD4) (PDB ID: 1DD1). The target protein structures were obtained from the Research Collaboratory for Structural Bioinformatics Protein Data Bank (RCSB PDB). The proteins of interest in the TGF-β/SMAD signaling pathway, which originated from X-ray crystallographic structures [41,42], were prepared for molecular docking.

All protein files were downloaded in “.pdb” format. The target protein structures were adjusted to a monomeric form by removing all non-protein atoms using the UCSF Chimera program. The processed structures were subsequently saved as “.pdb” files for docking analysis. The chemical structure of OLA (PubChem CID 5283387) was obtained from PubChem [43].

Protein–ligand blind docking was conducted using the SwissDock platform (http://www.swissdock.ch, accessed on 1 December 2023). The SwissDock server, which is built on the EADock DSS protein–ligand docking software available online: http://old.swissdock.ch (accessed on 12 March 2025) developed by the Swiss Institute of Bioinformatics (SIB) [44], was used to perform in silico docking, while UCSF Chimera was utilized for the visualization and analysis of docking results.

Prior to docking, the protein targets and OLA were pre-processed using the DockPrep tool in UCSF Chimera alpha software (version 1.18) [45]. Following docking, the interactions between the target proteins and the ligand were visualized in 2D using BIOVIA Discovery Studio Visualizer (version 21.1.0.20298, Waltham, MA, USA).

### 4.8. Western Blot Analysis

Cells were treated and incubated as outlined in Figure 1e. Following incubation, the cells were lysed using ice-cold RIPA buffer (Bio Basic Inc., Amherst, NY, USA) supplemented with protease and phosphatase inhibitor cocktails (Thermo Fisher Scientific, Waltham, MA, USA) for 30 min. The lysates were centrifuged at 12,000 rpm for 30 min at 4 °C, and the resulting supernatants were collected. Protein concentration was determined using the Bicinchoninic Acid Kit (BCA Kit). Equal amounts of protein samples were resolved on a 12% SDS–polyacrylamide gel (PAGE) and transferred to a 0.2 µm polyvinylidene fluoride (PVDF) membrane (Bio-Rad Laboratories, Inc., Hercules, CA, USA). The membrane was blocked overnight at 4 °C with 5% bovine serum albumin (Capricorn Scientific GmbH, Hesse, Germany) prepared in Tris-buffered saline with Tween 20 (TBST). The membrane was incubated with primary antibodies against alpha-smooth muscle actin, p-SMAD2/3 and SMAD2/3 (Cell Signaling Technology, London, UK) at room temperature for 1 h. After washing with TBST, it was incubated for an additional hour at room temperature with a horse radish peroxidase (HRP)-linked anti-rabbit IgG secondary antibody (Cell Signaling Technology, London, UK). Protein bands were visualized using a chemiluminescence substrate after a 5 min incubation and imaged using the ChemiDoc XRS+ Imaging System (Bio-Rad Laboratories, Inc., Hercules, CA, USA). Protein intensity was quantified with Image Studio Lite software Version 4.0.1. (LI-COR Corporate, Lincoln, NE, USA).

### 4.9. Data and Statistical Analysis

All in vitro experimental procedures were performed in triplicate. Data are presented as the mean ± standard deviation (SD), unless otherwise indicated. Statistical comparisons between group means were conducted using one-way analysis of variance (ANOVA), followed by Tukey’s post hoc test for multiple comparisons, as implemented in GraphPad Prism software (version 8.0.1). A *p*-value of < 0.05 was considered indicative of statistical significance.

## 5. Conclusions

This study demonstrates that oleamide (OLA), identified from *Moringa oleifera* Lam. (MO) leaves, effectively inhibits the activation of hepatic stellate cells and fibrosis: it suppresses TGF-β1-induced SMAD2/3 phosphorylation and SMAD4 expression, thereby disrupting the TGF-β/SMAD signaling pathway. OLA also downregulates fibrotic markers (e.g., ACTA2, COL1A1, COL4A1, TIMP-1, MMP-2, MMP-9) and reduces pro-inflammatory cytokines (IL-6 and IL-8). Molecular docking provides insights into the interactions between OLA and key SMAD pathway components, with favorable binding energies. These findings establish OLA as a promising natural therapeutic agent for mitigating hepatic fibrosis by targeting fibrosis-associated signaling pathways.

## Figures and Tables

**Table 1:** The affinity of OLA binding on target proteins in the SMAD signaling pathway.

PDB ID	Protein Name	Binding Free Energy (kcal/mol)	Interacting Amino Acid
6YIB	Mothers against decapentaplegic homolog 3	−8.27	ALA24, GLU20, ASP21, VAL95, GLU91
6YIA	Mothers against decapentaplegic homolog 2	−7.57	LEU229, TRP230, VAL178, Leu174, LEU222, ARG56, ARG129, GLU182
1DD1	Mothers against decapentaplegic homolog 4	−7.42	GLU417, GLY419, ALA421, PRO422, ARG416, ARG420, ASP415, TYR412, TYR413, GLN446, GLN442, PHE438, CYS443, LEU498, ASP439
5E8X	Transforming growth factor-beta (TGF-β) receptor type 1	−7.16	THR74, TYR75, THR73, SER33, VAL32, VAL34, VAL41, ILE85, GLU36, GLU86, HSD43, LYS84, PHE13
5E8V	Transforming growth factor-beta (TGF-β) receptor type 2	−6.99	LYS260, THR273, PHE327, VAL274, ALA329, ASN389, LYS330

## Data Availability

The data presented in this study are available within the article.

## Supplementary Materials

The following supporting information can be downloaded at https://www.mdpi.com/article/10.3390/ijms26073388/s1.
