# Supplementary material for: Anti-Fibrotic Effect of Oleamide Identified from the Moringa oleifera Lam. Leaves via Inhibition of TGF-β1-Induced SMAD2/3 Signaling Pathway
Source: Int J Mol Sci. 2025 Apr 4;26(7):3388. doi: 10.3390/ijms26073388 (PMC11989637; doi:10.3390/ijms26073388)
Supplement: Supplementary file 1 [file ijms-26-03388-s001.zip › ijms-3553782-supplementary.pdf]

Supplementary Material

Anti-fibrotic Effect of Oleamide Identified from the *Moringa oleifera* Lam. Leaves via Inhibition of TGF-β1-Induced SMAD2/3 Signaling Pathway

Chavisa Khongpiroon[1], Watunyoo Buakaew[2], Paul J. Brindley[3], Saranyapin Potikanond[4], Krai Daowtak[1] , Yordhathai Thongsri[1], Pachuenp Potup[1] and Kanchana Usuwanthim[1] *

*** Correspondence:** Kanchana Usuwanthim: Kanchanau@nu.ac.th

Preparation of *Moringa oleifera* (MO) leaves extract

The procedure for MO extraction and bioactive component identification was described in detail in our previous study [24]. Extraction of MO leaves involved sequential extraction with organic solvents of increasing polarity: hexane, ethyl acetate (EtOAc), and 95% ethanol (EtOH). MO powder (1 kg) was macerated in 1 L of hexane at room temperature for three days. The supernatant was filtered through Whatman filter paper No. 3, and the filtrate was concentrated using a rotary evaporator (Heidolph Hei-VAP Value HB/G3B) at 40°C under reduced pressure to obtain a semi-solid crude hexane extract. The residue was then macerated in 1 L of EtOAc at room temperature for three days, followed by filtration and evaporation to obtain the crude EtOAc extract. The same process was repeated using 1 L of 95% EtOH to obtain the crude EtOH extract. The extraction procedure was repeated three times for each solvent. The crude extracts were collected and stored at -20°C until used. For experiments, the extracts were dissolved in dimethyl sulfoxide (DMSO) and diluted in deionized (DI) water to a final concentration of 10 mg/mL (5% DMSO residual). The diluted extracts were filtered through a 0.2 µM filter (Millipore), aliquoted into microcentrifuge tubes, and stored at -20°C.

Fractionation of Crude MO Extract

The crude EtOAc extract (10 g) was dissolved in hexane-EtOAc (70:30) and fractionated using silica gel column chromatography with a gradient of hexane-EtOAc mixtures (90:10, 80:20, 70:30, 60:40, 50:50, 40:60, 30:70, 20:80, 10:90, and 100% EtOAc), followed by EtOAc-methanol (MeOH) (90:10, 80:20, and 100% MeOH) as the mobile phase. The collected fractions were monitored and combined based on their thin-layer chromatography (TLC) patterns. The active fraction (fraction no. 7; 135 mg) was further purified by silica gel column chromatography using a gradient of hexane-EtOAc (90:10, 80:20, 70:30, 50:50, and 100% EtOAc), followed by EtOAc-MeOH (90:10). The resulting sub-fractions (no. 7.1-7.8) were tested for anticancer activity using the MTT assay. The most potent sub-fraction (sub-fraction no. 7.7) was subjected to bioactive compound identification using LC-ESI-QTOF-MS/MS analysis. All extracts were stored at -20°C until use.

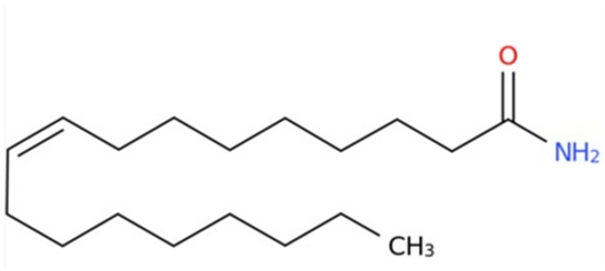

Figure S1. The structure of oleamide (OLA) or Cis-9,10-octadecenamide, one of the bioactive compounds identified in extracts of MO leaves.

Table S1. Primers for Real-time qRT-PCR

Genes	Description	Forward Primer (3′ → 5′)	Reverse Primer (3′ → 5′)
ACTA2	actin alpha 2, smooth muscle	CATCCTCATCCTCCCTTGAG	ATGAAGGATGGCTGGAACAG
COL1A1	Collagen type I alpha 1 chain	CCGGCTCCTGCTCCTCTTAGCG	CGTTCTGTACGCAGGTGATTGGTGG
COL4A1	Collagen type IV alpha 1 chain	CCTGGCTTGAAAAACAGCTC	CCCTGCTGAGGTCTGTGAAC
TIMP1	TIMP metallopeptidase inhibitor 1	CAAGATGTATAAAGGGTTCCAAGC	TCCATCCTGCAGTTTTCCAG'
MMP2	matrix metallopeptidase 2	AAGTATGGCTTCTGCCCTGA	ATTTGTTGCCCAGGAAAGTG
MMP9	matrix metallopeptidase 9	CGAACTTTGACAGCGACAAG	CACTGAGGAATGATCTAAGCCC
GAPDH	Glyceraldehyde-3-phosphate dehydrogenase	ATGACATCAAGAAGGTGGTG	CATACCAGGAAATGAGCTTG'
SMAD2	SMAD family member 2	TGCTCTGAAATTTGGGGACTGA	GACGACCATCAAGAGACCTGG
SMAD3	SMAD family member 3	ATCGTGAAGCGCCTGCTG	CATCCAGGGACCTGGGGA
SMAD4	SMAD family member 4	GCCCGAGCCCAGGTTATC	ACAATGCTCAGACAGGCATCA
